# Music across the love-span: a mixed methods study into the use of music in romantic relationships

**DOI:** 10.12688/openreseurope.19016.1

**Published:** 2024-12-24

**Authors:** Julia Vigl, Joshua S. Bamford, Abbigail Fleckenstein, Suvi Saarikallio

**Affiliations:** 1 Psychologie, Universitat Innsbruck Fakultat fur Psychologie und Sportwissenschaft, Innsbruck, Tyrol, 6020, Austria; 2 Department of Music, Art and Culture Studies, University of Jyväskylä, Jyväskylä, Central Finland, 40014, Finland; 3 Centre of Excellence in Music, Mind, Body and Brain, Jyväskylä, Finland, 40014, Finland; 4 Centre for the Study of Social Cohesion, University of Oxford School of Anthropology and Museum Ethnography, Oxford, England, OX26PE, UK; 5 Department of Psychology; RITMO Centre for Interdisciplinary Studies in Rhythm, Time and Motion, University of Oslo, Oslo, Norway, 0316, Norway

**Keywords:** Music, romantic relationships, social bonding, mate selection, compatibility, attractiveness

## Abstract

**Background:**

Music is known to be a powerful tool for social bonding, but its role in romantic relationships remains poorly understood. The present study investigated the relevance of music to three core aspects of love (intimacy, passion and commitment) across relationship stages: attraction, building, and maintenance.

**Methods:**

Using a mixed-methods approach, 174 participants responded to self-report ratings assessing the role of music for the three aspects of love across the three relationship stages, as well as open-ended questions reflecting on their music-related experiences at each relationship stage.

**Results:**

Results from quantitative ratings showed that music promotes intimacy and passion, particularly during the attraction and building phases, with less impact on commitment and during the maintenance phase. Participants with greater musical expertise and reward reported greater overall importance of music, suggesting that musical ability and preference may be used as a tool to assess compatibility between partners, rather than being generally attractive traits. Qualitative analysis of 351 coded open responses revealed a set of 55 key musical actions (e.g., listening, sharing and making music) and outcomes (e.g., bonding, (re)connecting and assessing compatibility) related to participants’ use of music throughout the three relationship stages. Themes such as signalling attraction and emotional communication were most prominent in the attraction phase, whereas bonding through shared musical activities was more common in later phases.

**Conclusion:**

The present study provides a first systematic investigation of the role of music for different phases and aspects of romantic relationships. The findings provide valuable insights for music research, relationship studies, and therapeutic practice, highlighting the role of music in fostering connection and intimacy in romantic relationships.

## Plain language summary

Music plays an important role in relationships, especially in the early stages of getting to know someone and building a connection. It can help people express identity, share emotions and bond through shared experiences. But how does music contribute to romantic love at different stages of a relationship, namely initial attraction, building a relationship and maintaining it over time?

This study asked people to rate how important music was at different stages of a relationship, and about the ways they used music in their relationships. It was found that music is most important for fostering intimacy and passion in the early stages, but has less impact on commitment in long-term relationships. People with greater musical ability or enjoyment of music valued it more in their relationships, suggesting that music may signal compatibility rather than simply attractiveness. Participants' personal stories revealed that music-related activities, such as listening to, sharing or creating music together, helped couples to connect and communicate. For example, in the attraction phase, music is often used to signal compatibility or express feelings, while in later phases can be used to bond through shared activities.

This research highlights the power of music in romantic relationships and offers new insights into how couples use music to foster connection and intimacy throughout their shared journey.

## Introduction

Music plays an important role in everyday life and serves not only personal purposes (e.g. mood regulation, evoking positive memories or helping with event processing), but also social functions (e.g., Dingle *et al*., 2021; Tarr *et al*., 2014). For example, musical preferences are among the most frequent topics of conversation when strangers initiate relationships ( Rentfrow & Gosling, 2006), and social bonding was found to develop more rapidly in singing groups than in those engaged in crafts or creative writing ( Pearce *et al*., 2015). While most research to date has concentrated on music's social functions in contexts involving strangers or friends, a recent comprehensive theoretical review by Bamford *et al*. (2024) explored the potential role of music in the specific case of romantic relationships, addressing mate selection and social bonding functions of musical experiences over different relationship stages. Building on this work, the aim of the present study was to use a combination of quantitative and qualitative data to explore the role of music at different stages of romantic relationships and to examine which musical activities and their outcomes influence partner choice and facilitate or inhibit romantic feelings.

### Aspects of romantic love

Romantic love has been widely portrayed in literature, film and music, leading to a common understanding of the concept despite different definitions. Key elements include longing for an enduring bond with a specific individual ( Hatfield & Rapson, 1993), strong emotional attachment combined with sexual desire ( Goode, 1959), and interdependence, in which partners influence each other's behaviours and decisions ( Bradbury & Karney, 2019). A prominent research framework is Sternberg's (1986) Triangular Theory of love, which identifies three key components: intimacy, passion, and commitment. Different levels of these components differentiate different forms of love, ranging from non-love (absence of all components) to consummate love (strong presence of all three components).

Within this framework, *intimacy* represents the 'warm' components of love, involving mutual interactions and self-disclosure, deep affective feelings, and an inclination towards and pursuit of closeness ( Sternberg, 1986). *Passion* embodies the 'hot' component of love and includes cognitive (e.g., persistent thoughts), affective (e.g., sexual attraction and intense emotions), and behavioural (e.g., seeking physical closeness) aspects ( Hatfield & Sprecher, 1986; Sternberg, 1986). The third component, *commitment*, is the 'cold' component of love, involving the decision to enter into and maintain a long-term relationship and behaviours aimed at achieving this goal, such as devaluing alternatives, making sacrifices for the partner, and accommodating negative partner behaviour ( Sternberg, 1986).

The triangle theory of love has significantly influenced subsequent theories of romantic love, has been empirically validated (e.g., Acker & Davis, 1992; Lemieux & Hale, 2002), and shows potential universality across cultures ( Sorokowski *et al*., 2021).

### Romantic relationship stages

According to Levinger's (1980) concept of long-term relationships, as adopted in the theoretical framework of Bamford *et al*. (2024), three primary stages of romantic relationships can be distinguished: attraction, relationship building and, if there is no deterioration or termination, a maintenance phase. These stages align well with other influential models of relationship development, such as the Staircase Model ( Knapp, 1978), Social Penetration Theory ( Altman & Taylor, 1973), and Uncertainty Reduction Theory ( Berger & Calabrese, 1975).

During the *attraction stage*, potential partners get to know each other, leading to emotional and cognitive evaluations of each other. The emergence of attraction is influenced by a variety of internal and external factors, including personality traits, values, physical appearance, mutual liking, and perceived similarities in personality, values, and attitudes (e.g., Buss & Barnes, 1986; Luo, 2017; Montoya & Horton, 2020). Communication at this stage is typically superficial and guided by social norms, with limited personal disclosure (e.g., Berger & Calabrese, 1975; Knapp, 1978)

The phase of *building a relationship* is characterised by a rapid increase in self-disclosure, allowing for deeper communication and the sharing of personal opinions and attitudes (e.g. Altman & Taylor, 1973). As communication intensifies and bonds strengthen, partners may form a relational identity, make the relationship public, or take significant steps such as marriage ( Knapp, 1978).

Once partners define themselves as couples or romantic partners, the relationship process continues into the *maintenance phase*. Individual strategies for maintaining the relationship may include positive illusions, idealisation, and expressions of gratitude, while interactive strategies include effective communication, conflict management, dyadic coping, mutual support, humour, and engaging in joint activities ( Ogolsky *et al*., 2017).

The relative importance and prominence of the three components of the triangle of love - intimacy, passion, and commitment - changes at different stages of a relationship. The attraction phase (which typically lasts up to six months) is characterised by a rapid increase in passion for a potential partner. As the relationship moves into the building phase, and for about the first four years, couples experience increased intimacy, with a slight decrease in passion. Beyond this period, commitment becomes more central, with intimacy remaining at medium to high levels, while passion continues to decline ( Acker & Davis, 1992; García, 1998; Wojciszke, 2002).

### Music for mate choice and social bonding

The potential for music to foster social connection has been widely studied; however, most studies focussed on groups of strangers or friends, but not couples. For example, musical preferences are important expressions of identity (e.g., Shepherd & Sigg, 2015) and are often used to initiate conversations with strangers ( Rentfrow & Gosling, 2006). Having similar tastes can strengthen in-group bonds and encourage the development of friendships ( Lonsdale & North, 2009; Selfhout *et al*., 2009). Singing together elicits sociobiological bonding responses ( Bowling *et al*., 2022; Kreutz, 2014), and compared to groups engaged in other activities, singing has been shown to be associated with accelerated bonding processes ( Pearce *et al*., 2015). Collective musical activities, such as drumming and improvisation, were further associated with prosocial behaviour, feelings of belonging, and commitment ( Kokal *et al*., 2011; Kokotsaki & Hallam, 2007; Verneert *et al*., 2021). These effects are often attributed to the synchronisation involved, as engaging in synchronised actions commonly leads to increased feelings of social affiliation, trust, and prosocial behaviour ( Bamford *et al*., 2023; Hove & Risen, 2009; Mogan *et al*., 2017; Rennung & Göritz, 2016; Vicaria & Dickens, 2016). Music provides a temporal scaffolding that makes it easier to coordinate actions in time ( Tarr, 2017), while also creating a social space that aligns the intentions of participants ( Cross, 2014).

 However, while there is substantial research on the capacity of music to foster bonding between groups or strangers, there is little work specifically looking at how music might influence partner choice or enhance feelings of intimacy, passion, or commitment in romantic relationships. One couple-specific phenomenon is the 'couple-defining song'. According to Harris *et al*. (2020), 60% of couples have songs they associate with their relationships. These songs are associated with greater relationship intimacy and may help to foster a sense of "we", while also aiding in the storage and retrieval of positive autobiographical memories.

Other research has focused on attractiveness and has shown that creativity is generally considered an attractive trait in mate choice (e.g., Karamihalev, 2013), with some studies specifically highlighting musical traits - such as being a musician, improvisational skills, or dance ability - as attractive qualities ( Hagen & Bryant, 2003; Madison *et al*., 2018; Marin & Rathgeber, 2022). It has been argued that musicality is generally attractive because it indicates general fitness ( Miller, 1999). However, other studies have found no significant associations between musical ability and mating success ( Mosing *et al*., 2015) or suggested that such associations are only present among individuals who are highly musical themselves ( Bongard *et al*., 2019).

In terms of relationship maintenance, couples often report engaging in musical activities together ( Campbell *et al*., 2011). However, to the best of our knowledge, only one study has specifically examined the effects of engaging in dyadic musical activities, such as listening to music together, sharing and exploring music, or making music as a couple. This study found that even when controlling for other non-musical dyadic activities, couples who engaged in dyadic musical activities (as opposed to structured group activities with others) experienced higher levels of commitment, mediated by interpersonal coordination and self-disclosure ( Harwood & Wallace, 2021).

Overall, research on how music affects different aspects of romantic love at different stages of relationships is limited. It remains unclear how couples use music in their relationships, whether and how these actions influence love-related outcomes, and which individuals are more or less influenced by music during the development and maintenance of romantic feelings.

### The present study: Aims and Research Questions

The present study aimed to empirically test the role of music in romantic relationships as proposed by Bamford *et al*. (2024). We used a mixed-methods design that employed both quantitative and qualitative questions. The following three research questions guided this study:

*RQ1*: How important is music in the context of romantic relationships, and does it contribute to strengthening intimacy, passion, and commitment at different stages of romantic relationships?

    According to the model outlined by Bamford *et al*. (2024), we expect that the role of music for passion should be most important in the attraction phase, intimacy to be most important in the building phase, and commitment to be most important in the maintenance phase. However, these predictions are based on limited prior research.

*RQ2*: Which personal characteristics are associated with a greater role for music in romantic relationships?

    Here there are two contrasting hypotheses. On the one hand, people often seek similarity in romantic partners ( Luo, 2017), so musicality may correlate with the perceived importance of music. On the other hand, musicality may be generally attractive because it indicates general fitness ( Miller, 1999), and therefore music should be equally important for all participants.

*RQ3*: What is the nature of music-related experiences that individuals recall in the context of romantic relationships across three relationship phases?

While RQ1 and RQ2 will be answered with quantitative data, RQ3 is purely exploratory and will be analysed qualitatively with data-driven template analysis using participants' open responses.

## Methods

### Participants

A total of 174 individuals (out of 402 individuals that visited or started the survey) completed all parts of the study (145 females, 25 males, 3 non-binary, 1 other), with a mean age of 27.18 years ( *SD* = 9.27, range = 18–63). Most participants reported Finland ( *n* = 49), Austria ( *n* = 40), and Germany ( *n* = 38) as their nationalities; the other nationalities reported by more than one participant were Italy (18), the United Kingdom (4), and Australia (4). On average, the participants were highly educated, with 56% having a university degree and 34% having a high school diploma.

Regarding musical background, 61% of the sample had been playing an instrument or singing for an average of 15.80 years ( *SD* = 11.20, range = 0–55) and about 3.41 hours per week ( *SD* = 4.40, range = 0–24). Slightly more than half of the sample identified themselves as non-musicians (57%), 35% as amateurs or serious amateur musicians, and 8% as (semi-)professional musicians.

 Most participants were in a romantic relationship at the time of participation ( *n* = 118, 68%); 36 (21%) were single, and the remaining 20 (11%) were in the dating stage. Of the participants who were not single ( *n* = 13), 116 (84%) were in the maintenance phase of a relationship, 13 (9%) were in the building phase, and 9 (7%) were in the initial attraction phase. Participants in romantic relationships had been together with their partner for a mean of 5.28 years ( *SD* = 2.38, range = 0.08 - 40.42).

### Procedure

The study was conducted as an online survey using the open access software LimeSurvey ( Limesurvey GmbH, 2022). Participants were recruited through email invitations sent to all students at the University of Innsbruck (Austria) and the University of Jyväskylä (Finland). In addition, the study invitation was shared on social media platforms, including Facebook and Instagram. The only inclusion criterion was that participants had to have experienced at least one romantic relationship of any kind (e.g. monogamous, polyamorous, open, heterosexual, homosexual or bisexual) at some point in their lives. After giving informed consent and completing demographic questions, participants were explained the three relationship phases (attraction, building and maintenance) and aspects of love (intimacy, passion, and commitment). They were then asked to provide qualitative information about their experiences with music in the context of romantic relationships, answering quantitative and qualitative questions separately for each phase. Finally, participants provided information about their musical background, current relationship status, music-related reward experiences, and answered additional questions about the importance of music in the context of love. The survey was distributed on 12 pages with approximately 3-10 questions per page. The data collection took place between November 2023 and February 2024.

### Quantitative measures

*Role of music in romantic relationships*. Separately for each of the relationship stages (attraction, building, maintenance), we first asked participants how they would rate the overall importance of music (“ *At this stage of the relationship, is/was music important to you?*”) on a 5-point scale (1 = *not at all*, 5 = *totally*). In addition, and using the same answer format, we assessed if and how much music contributed to strengthening the three love components according to the Triangle Theory of Love ( Sternberg, 1986), namely passion (“ *At this stage of the relationship, did music contribute to strengthening the passion you feel towards someone?*”), intimacy (“ *At this stage of the relationship, did music help you to feel more connected to someone?*”), and commitment (“ *At this stage of the relationship, did music contribute to the decision to continue the relationship with someone?*”). At the end of the survey, we asked a few more questions about the integration of music in participants' relationships (all on a 5-point scale; 1 = *No, not at all*, 5 = *Yes, totally*): If they talk about music with (potential) partners, if a (potential) partner's musical taste and musical ability is important to them, if they ever had a song that represented the relationship ("our song"), and if they think musical compatibility is more important than compatibility in other areas of leisure.

*Musical Expertise*. To assess participants' musical background, we used a single item from the *Ollen Musical Sophistication Index* ( Ollen, 2006) on self-reported musical status (1 = *non-musician*, 2 = *music-loving non-musician*, 3 = *amateur musician*, 4 = *serious amateur musician*, 5 = *semi-professional musician*, 6 = *professional musician*), supplemented by self-generated questions on whether they played an instrument or sang, for how many years, and how many hours per week they practised. To adjust for age differences, we re-coded years of practice to indicate the percentage of life spent playing an instrument or singing (years of practice/age). As these four items were internally consistent, we z-transformed them and calculated a continuous composite score for musical expertise (α = 0.86).

*Music Reward*. Music-related rewards were assessed using the *Barcelona Music Reward Questionnaire* (BMRQ; Mas-Herrero *et al*., 2013). It assesses different facets of music reward and had high internal consistency in our sample: music-seeking (α = 0.61; e.g., “ *I inform myself about music I like*”), emotion evocation (α = 0.63; e.g., “ *I sometimes feel chills when I hear a melody that I like*”), mood regulation (α = 0.70; e.g., “ *Music calms and relaxes me*”), sensory motor (α = 0.73; e.g., “ *Music often makes me dance*”), social reward (α = 0.54; e.g., “ *Music makes me bond with other people*”), and an overall musical reward score (α = 0.82). All items were measured on a 5-point scale (1 = *strongly disagree*, 5 = *strongly agree*). As we are not aware of a validated German version of the BMRQ, three bilingual translators independently translated the scale into German and selected the best translations for all items.

*Demographic and relationship questions.* In addition to demographic information such as age, gender, education, and nationality, we assessed whether participants were in a romantic relationship, and if so, at what stage (attraction, building, maintenance) and for how many years.

The exact wording of all the questions, together with the labels for the answers, can be found in the OSF repository (section quantitative data).

### Qualitative measures

For each of the three relationship phases (attraction, building, and maintenance), we provided participants with an empty text box to list their experiences with music that were relevant to their romantic relationships at that stage. The wording was:

*“Please list any experiences with music that have been relevant to the relationship during the phase of [attraction/building/maintaining] a relationship. These can be positive activities that have strengthened the relationship, or negative experiences that have contributed to drifting apart”.*

### Data analysis

***Quantitative analysis.*** We conducted quantitative analyses to address the first two research questions concerning participants' perceptions of the role of music in romantic relationships and its association with personal characteristics. Two repeated measures ANOVAs assessed whether the importance of music varied across relationship stages or love aspects, applying Greenhouse-Geisser corrections where necessary. The first analysis predicted the role of music based on aspects of love, relationship stage, and their interactions. The second analysis predicted the overall importance of music based on the relationship stage. Pearson’s correlations were used to identify the associations between personal characteristics and the role of music in romantic relationships.

***Qualitative analysis.*** We used template analysis ( Brooks *et al*., 2015; Crabtree & Miller, 1992) to analyse participants' responses to three open-ended survey questions about their experiences of music in romantic relationships. Following the methodology outlined by Brooks *et al*. (2015), we first created a collaborative template among all authors. The template for the current analysis was inductively defined based on empirical observations from the initial screening of responses. The initial template delineated two main categories: 'action', which included activities such as making, sharing and listening to music, and 'outcome', which included signalling attraction or compatibility, bonding and regulating emotions and affect. Once a basic code structure was established, two authors independently and parallelly coded all responses, meeting periodically to refine or expand the template, as needed. Each answer could be assigned multiple codes. Ultimately, we arrived at a final set of 55 codes organised into four second-level categories for action and five for outcome. An overview of the codes, higher-order themes, and dimensions identified is shown in Figure 2, and Table S3 (see OSF repository) provides the descriptions of all codes. Inter-rater agreement was assessed using Cohen's kappa coefficient ( McHugh, 2012).

## Results

### Quantitative results

**Figure 1.**
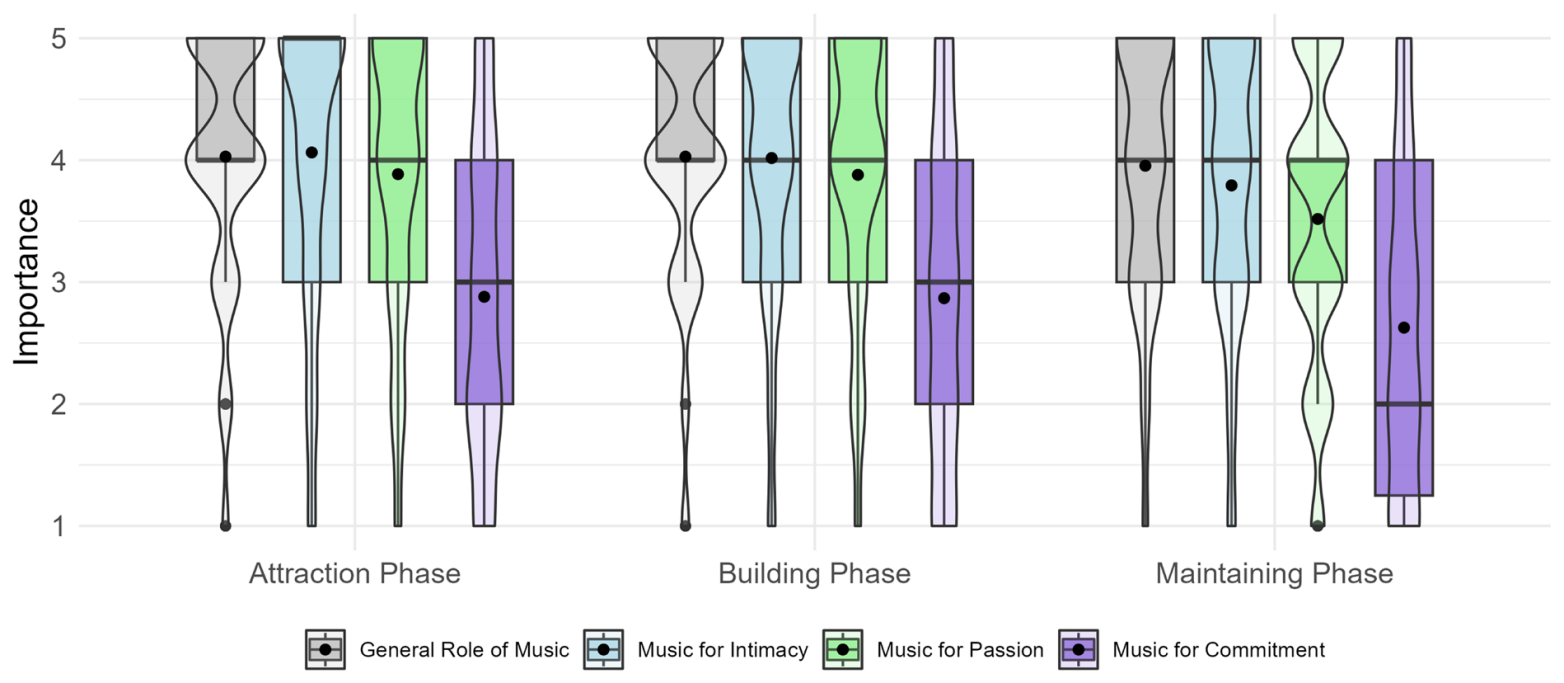
The importance of music across the three phases of romantic relationships. *Note.* A combination of boxplots and violin plots is used to visualise the data distributions. Mean values are indicated by black circles. The importance of music was measured on a scale from 1 = *not at all* to 5 = *totally*.

To compare the importance of intimacy, passion, and commitment across the three relationship phases (RQ1), we performed a repeated-measures ANOVA with aspect and phase as within-subject factors predicting the importance of music. This analysis revealed significant main effects for both aspect, *F*(1.39, 240) = 195.75, *p* < .001, ηp² = 0.53, and phase, *F*(1.62, 280) = 13.44, *p* < .001, ηp² = 0.07, and no interaction effect, *F*(3.56, 616) = 0.87, *p* = .475, ηp² = 0.01. Pairwise comparisons using Bonferroni correction indicated significant differences between all relationship aspects ( *p* < .001). Regarding relationship phases, there were no differences between the attraction and building phases ( *p* = 1.00), but there were differences between the maintenance phase and both the attraction and building phases ( *ps* < .001). The perceived general role of music did not differ across relationship phases, *F*(1.85, 319.2) = 0.77, *p* = .462, ηp² < 0.01. As shown in Figure 1, participants reported a high general role of music in all three phases ( *M* = 4.00, *SD* = 0.89). The importance of music for the three components was higher in the attraction ( *M* = 3.61, *SD* = 1.08) and building ( *M* = 3.59, *SD* = 1.02) phases than in the maintenance phase ( *M* = 3.31, *SD* = 1.05), while it appeared to be most important for intimacy ( *M* = 3.96, *SD* = 0.97), followed by passion ( *M* = 3.76, *SD* = 0.99), and commitment ( *M* = 2.79, *SD* = 1.18).

To determine whether there are personal characteristics associated with the role of music in romantic relationships (RQ2), we calculated correlations between the role of music in general and for the three aspects aggregated across phases with the assessed personal characteristics: demographics (gender, age, education), musical expertise, and music reward experiences. As detailed in Table 1, of the characteristics assessed, music reward experiences were associated with all outcomes, whereas musical expertise was correlated with the general role of music.

**Table 1.** Correlations between the importance of music and assessed characteristics including means and standard deviations.

Variable	M (SD)	1	2	3	4	5	6	7	8
1 Role of music overall	4.00 (0.89)								
2 Importance of music for passion	3.76 (0.99)	.73 ***							
3 Importance of music for intimacy	3.96 (0.97)	.71 ***	.88 ***						
4 Importance of music for commitment	2.79 (1.18)	.58 ***	.63 ***	.61 ***					
5 Musical expertise	0.00 (0.83)	.24 **	.11	.08	.14				
6 BMRQ Total Score	4.04 (0.46)	.50 ***	.44 ***	.40 ***	.24 **	.28 ***			
7 Gender::female	0.83 (0.37)	.05	.00	.05	-.02	-.01	.12		
8 Age	27.18 (9.27)	.07	-.02	.00	.13	.14	-.14	-.07	
9 Education	4.77 (1.17)	-.04	-.07	-.06	.04	.14	-.13	.08	.50 ***

*p < .05, ** p < .01, ***p < .001

In more detail, Table S1 in the Extended Data (see OSF repository) outlines the correlations between musical expertise and each BMRQ subscale across different relationship phases and their respective outcomes. This analysis suggests that musical expertise is specifically associated with higher importance of music in the attraction and building phases, but not in the maintenance phase. Moderate correlations were observed between all BMRQ scales and outcomes, with the BMRQ Social Reward subscale showing the strongest associations, while associations with the BMRQ Sensory Motor subscale appeared to be somewhat weaker.

In response to additional 5-point scale questions, participants indicated that they typically discussed their musical preferences with their (potential) partners ( *M* = 4.28, *SD* = 0.86) and placed moderate importance on their partner's musical taste ( *M* = 3.52, *SD* = 1.06), but less importance on their partner's musical ability ( *M* = 2.19, *SD* = 1.14). On average, participants tended to have songs that they considered defining their relationships with ( *M* = 3.70, *SD* = 1.37). Overall, the participants did not perceive musical compatibility as more important than compatibility in other leisure activities ( *M* = 2.54, *SD* = 1.05). Consistent with findings regarding the role of music in romantic relationships, higher scores on these additional questions were associated with higher levels of musical expertise and musical reward (see Table S2 in the OSF repository for more details).

### Qualitative results

**Figure 2.**
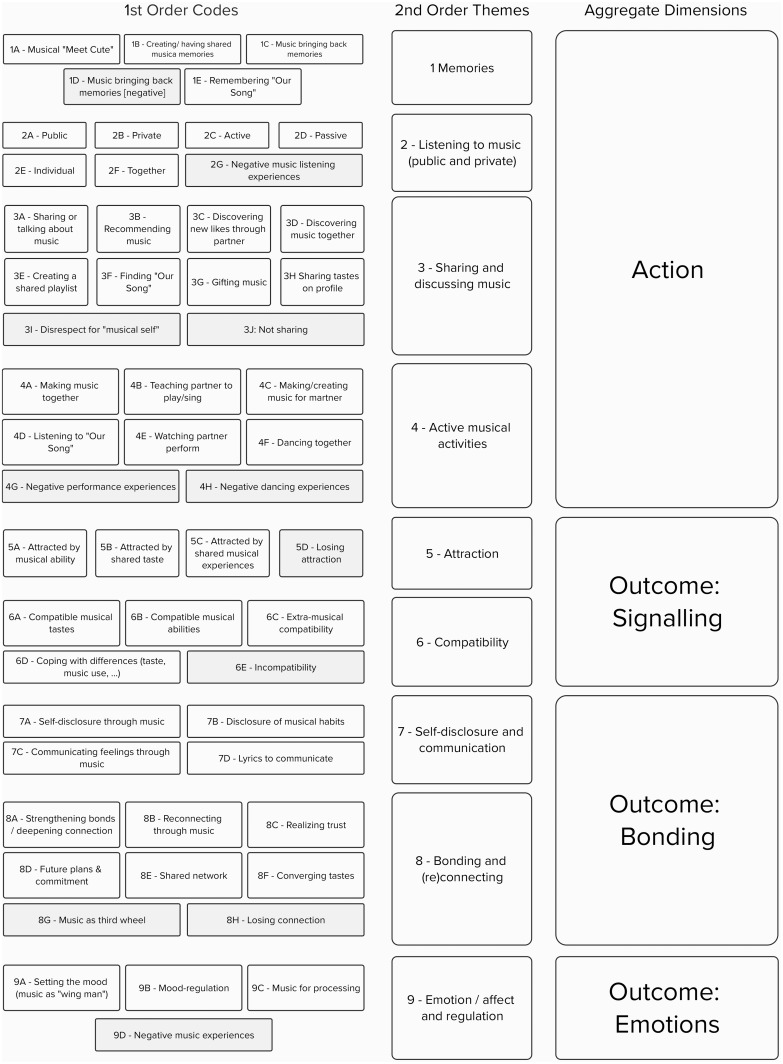
Full list of codes and higher order themes. Note. Negative valence codes are shown in grey. Code descriptions can be found in the extended data (see OSF repository).

Out of 174 participants, 129 provided responses on musical experiences in the attraction phase, 119 in the building phase and 103 in the maintenance phase, resulting in 351 coded responses. Qualitative template analysis, as described in the Methods section, yielded 55 distinct codes grouped into nine higher order themes (memories, listening to music, sharing and discussing music, active musical activities, attraction, compatibility, self-disclosure and communication, bonding and (re)connecting, emotion/affect, and regulation) and two aggregate dimensions (action and outcome), as shown in Figure 2.

Inter-coder reliability was substantial, with κ = 0.62, z = 104, p < .001 across all codes and phases. Agreement was similarly high for the attraction (κ = 0.62, z = 60.27, p < .001), building (κ = 0.60, z = 58.65, p < .001), and maintenance (κ = 0.63, z = 61.65, p < .001) phases. Tables S4 and S5 in the Extended Data (see OSF repository) further show that all Kappas for second-order themes were consistently above 0.40, indicating at least moderate agreement (M = 0.57, SD = 0.12, range = 0.40 - 0.65). Of the 55 first-order codes, 76% showed at least moderate agreement (> 0.40), and 96% achieved at least fair agreement (> 0.20) (M = 0.58, SD = 0.21, range = 0.12 - 1.00).

Given the high level of agreement between the coders, we present the qualitative responses based only on those codes on which both coders agreed, to increase the robustness of the findings. Consequently, only the codes that both coders identified as present in a participant's response were included in the analysis.

***Aggregate dimensions: Action and Outcome.*** Regarding the two aggregate dimensions of action and outcome (the latter subdivided into bonding, signalling, and emotions), 72.3% of the identified codes corresponded to musical actions, which included activities such as listening to music, sharing music, and creating music. Bonding as an outcome, involving (re)connection and self-disclosure, was present in 12.6% of the responses, signalling as an outcome, indicating attraction or compatibility, in 11.8%, and emotion as an outcome, including (co)regulation of emotions in terms of setting the mood and processing emotional experiences, in 3.3%.

Across all relationship phases, musical actions show a consistent presence, accounting for 68.6%, 73.0%, and 75.7% in the attraction, building, and maintenance phases, respectively. Similarly, bonding was observed across all phases with 10.3%, 14.0% and 13.7% in the attraction, building and maintenance phases respectively. Signalling was most pronounced in the attraction phase (16.5%), followed by the building phase (9.6%), and maintenance phase (8.9%). Finally, emotions were less pronounced in all phases, with 4.7%, 3.4%, and 1.7% in the attraction, building, and maintenance phases, respectively.

**Figure 3.**
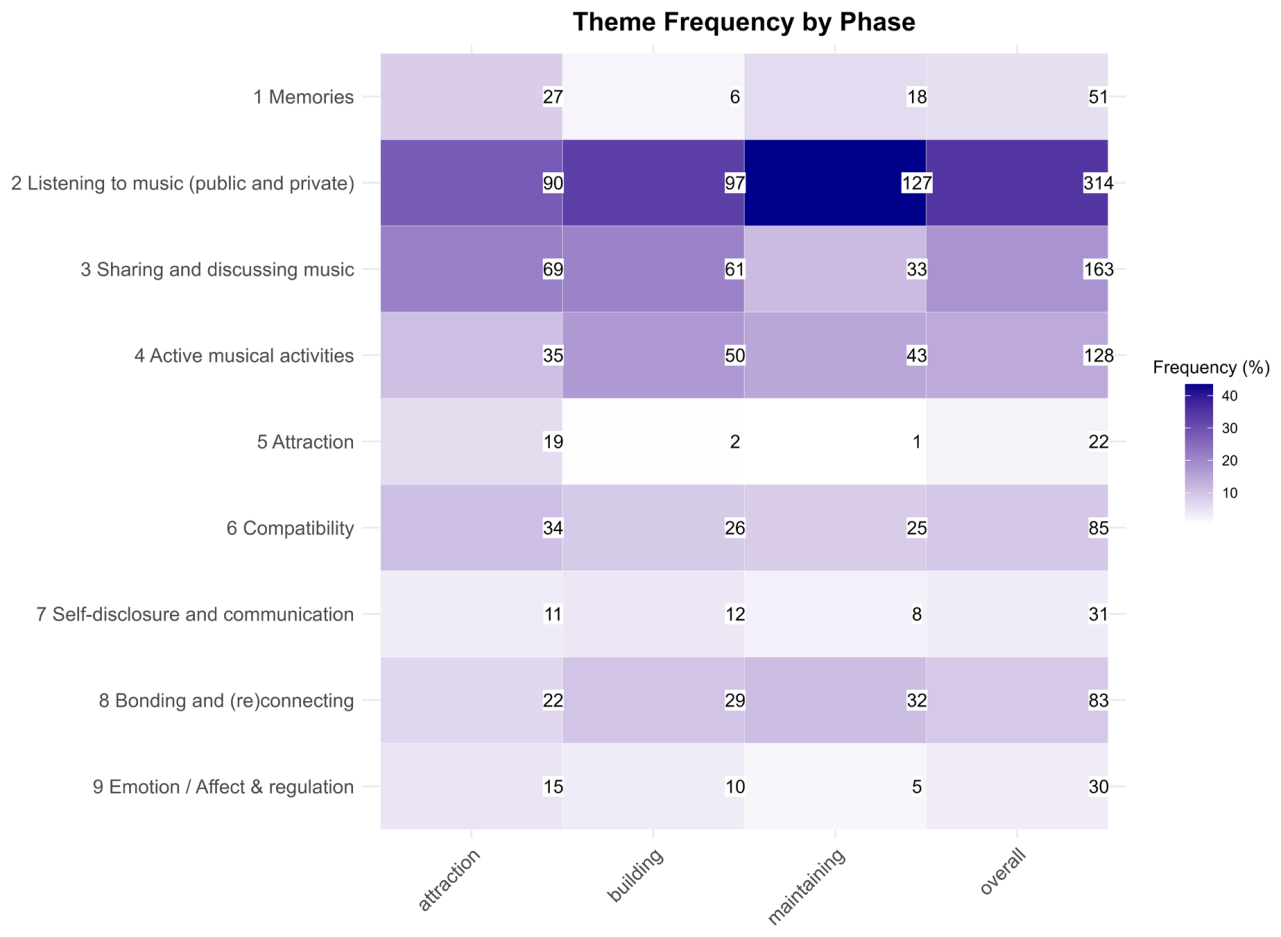
Frequencies of second-order themes across relationship phases.

***Second-order themes.*** The heatmap in Figure 3 provides an overview of theme frequencies overall and across phases. Overall, the most frequent theme was listening to music in public and private contexts, followed by sharing and discussing music and engaging in active musical activities. We also observed variations in the prominence of themes across phases. For example, 'listening to music' increased in frequency from the attraction phase to the maintenance phase, while 'sharing and discussing music' was more common in the attraction and building phases and decreased in the maintenance phase. The theme of 'memories' appeared in all phases, but was most prevalent in the attraction and maintenance phases, where participants referred to creating shared memories early on and recalling them later. These participant responses include quotes such as, “Having that song as a reminder of all the good in the relationship and why you love the other person, having positive memories tied to that and other songs that are brought up when you listen to them[...]” (Participant 116), and

“Certain songs discovered and consumed during that period (especially when they were relationship pop [songs]) connect with that time and reinforce the feeling of attraction. I've always felt that the songs I've consumed a lot tie in with important events in my life during the listening period, as well as the seasons.” (Participant 168)

Feelings of attraction through music were the most prevalent in the attraction phase, whereas references to compatibility through music were consistently present in all phases. The themes 'Self-disclosure and communication' and 'Emotion / affect & regulation' were more prominent in the early stages of the relationship. Regarding self-disclosure, participants detailed instances in which they revealed personal information to their partners, including their musical preferences and experiences, and used songs or song lyrics to communicate their feelings and intentions to their partners. Quotes exemplifying this theme include, “we shared playlists of music that we like. also we exchanged playlists with music in our native languages, as we are an international couple (finnish and spanish).” (Participant 17), “You can also ask which songs you listened to as a child and find out something about your family environment.” (Participant 326), Regarding emotion / affect & regulation, participants wrote about experiences in which music was used either intentionally or unintentionally to set the mood or affective tone while together or independently. Examples of this theme in participants’ responses include quotes such as,

“We both have a playlist of songs that the other one has suggested to us. I've added ‘extra’ songs to mine that remind me of him/our relationship. And when I miss him or am generally just having a bad day or am sad, I listen to this playlist. It calms me down.” (Participant 386)

Many participant responses in which this theme was observed discussed the importance of music before, during, and/or after sex. These participants’ responses shared ideas such as:

“I think that the choice of music is extremely important during sex. In my experience, the type of music has a significant effect on sex and therefore also determines whether it vibes. What I've also noticed personally in this context is that "more relaxed" and "romantic" music only feels good with people you really like. So music can also be an indicator of how comfortable I feel with a person and how vulnerable I can be.”(participant 238)

***First-order codes.*** In the action dimension, three of the most frequent codes were related to listening to music: listening to music together (2F, *n* = 139, 15.3%), listening to music in public (2A, *n* = 71, 7.8%) and listening to music in private (2B, *n* = 48, 5.3%). In addition, sharing and talking about music was represented in 7.6% of the codes ( *n* = 69), and making music together in 5.1% of the codes ( *n* = 46). In the outcome dimension, only one code appeared in nearly five percent of the responses: compatible musical tastes (6A, *n* = 45, 4.96%). The second-most prevalent code was strengthening bonds/deepening connections (8A, *n* = 42, 4.63%).

Table 2 provides an overview of the top three codes in each relationship phase, separately for the action and outcome dimensions. Across all phases, the most common action codes consistently involve listening to music together, whereas the outcomes of musical activities vary according to the relationship phases. In the attraction and building phases, partners primarily focus on (in)compatibility, shared musical tastes, and deepening bonds through music. In the maintenance phase, coping with differences becomes more prominent, alongside topics such as incorporating music into future plans and commitments. Interestingly, negative experiences were more frequently mentioned in the building and maintaining phases, namely the realisation of incompatibility and loss of connection. Quotes from participants' responses exemplifying these negative experiences shared ideas such as,

**Table 2.** Most prevalent (top three) first-order codes across relationship phases.

Code	Frequency	Percentage (by phase)
***Attraction Phase***		
**Action**		
2F - Together (music listening)	40	12.4
3A - Sharing or talking about music	38	11.8
2A - Public (music listening)	17	5.3
**Outcome**		
6A - Compatible musical tastes	25	7.8
8A - Strengthening bonds / deepening connection	17	5.3
5A - Attracted by musical ability & 5B - Attracted by shared taste	8	2.5
***Building Phase***		
**Action**		
2F - Together (music listening)	46	15.7
3A - Sharing or talking about music	20	6.8
2A - Public (music listening) & 4A - Making music	19	6.48
**Outcome**		
8A - Strengthening bonds/deepening connection	17	5.8
6A - Compatible musical tastes	14	4.8
6E - Incompatibility	8	2.7
***Maintaining Phase***		
**Action**		
2F - Together (music listening)	53	18.2
2A - Public (music listening)	35	12.0
2B - Private (music listening)	22	7.53
**Outcome**		
6D - Coping with differences	12	4.11
8A - Strengthening bonds/deepening connection	8	2.7
6E - Incompatibility, 8D - Future plans & commitment, & 8H - Losing connection	7	2.4

“Different music preferences led to us growing apart (he never really wanted to listen to music, but it was important to me) - even with the songs we listened to, when we listened to music we became estranged (I think) because we only ever listened to his songs.” (Participant 370).

A more detailed heat of the frequency of all 55 first-order codes across the relationship phases is provided in Figure S1 in the Extended Data (see OSF repository).

**Figure 4.**
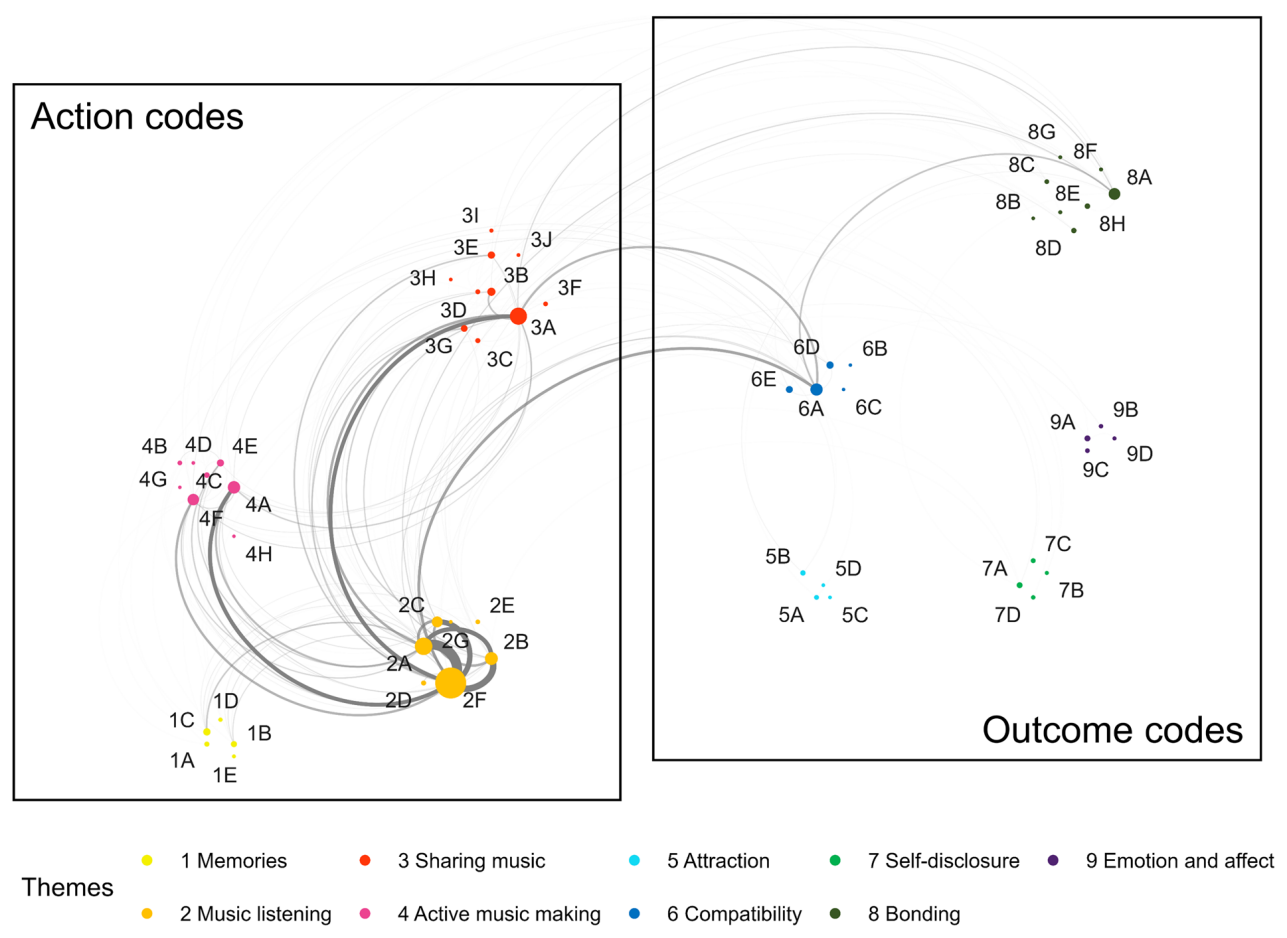
Co-occurrences of action and outcome codes across all relationship phases. *Note.* Circle sizes indicate the frequency of individual codes, and line thicknesses indicate how often the two codes were mentioned in the same responses.

***Co-occurrence of codes.*** To explore whether certain action and outcome codes were frequently mentioned together, we conducted a thematic co-occurrence analysis following the method outlined by Scharp (2021). As shown in Figure 4, outcome codes belonging to compatibility (Theme 6) and bonding (Theme 8) mostly co-occurred with action codes. Of the compatibility codes, compatible tastes (6A) showed the most co-occurrences, specifically listening to music codes (2F together, n = 19; 2C active, n = 7; 2A in public, n = 7), sharing music (3A, n = 15), and actively making music (4A, n = 5). Participant responses illustrating these co-occurrences included descriptions of actions such as, “Discussing musical preferences, and finding common interests, setting up a date location based on mutual musical interests (e.g. jazz bar), meeting up at a festival.” (Participant 110). The codes belonging to bonding (Theme 8), specifically strengthening bonds (8A), mostly co-occurred with listening to music (2F, n = 8) and sharing music (3A, n = 8). Responses such as, “Going to concerts together and generally listening to songs that both partners like was a nice experience. I feel more connected to my partner when he has at least some idea of what music I like [...].” (Participant 245- 8A and 2F) demonstrate the co-occurrence of these codes.

A full matrix of co-occurrences overall and separately by relationship phase can be found in the Extended Data (see OSF repository).

## Discussion

The present study is the first to explore the role of music for different aspects of love across the different stages of romantic relationships. The quantitative results suggest a medium to high overall importance of music, which is stronger for intimacy and passion than for commitment, and for the attraction and building phases than for the maintenance phase. In addition, music-related personal characteristics moderated the observed associations, with stronger correlations present in individuals with higher levels of musical expertise and music reward experience.

From the 351 qualitative responses, two aggregate dimensions (action and outcome) emerged, comprising nine second-order themes and 55 first-order codes. Participants frequently mentioned musical actions, with listening to music, sharing or discussing music, and making music together as the most common. Outcomes were less frequently mentioned, but centred on bonding, (re)connecting, and learning about compatibility through music. Notably, themes such as signalling attraction and compatibility and using music for emotional communication and regulation were most common in responses for the attraction phase, whereas bonding through music and engaging in musical activities were more commonly reported for the building and maintenance phase.

### Music across the love-span

Using a mixed-methods approach, the present study provides an overview of how important music is at different relationship stages, by enhancing different facets of love. The open-ended qualitative responses allowed participants to elaborate on the musical behaviours they associate with each stage and why they were important. Even though we have generated the codes purely data-driven, some parallels with Sternberg’s Triangular Theory can be drawn ( Sternberg, 1986). Most notably, participants often reported music as a bonding experience, which could correspond to an increased level of ‘intimacy’ in Sternberg’s model, while ‘attraction’ codes could correspond to the passion dimension. Codes relating to ‘commitment’ were less frequent, and overall music was rated as being least important for this facet, although some exceptions will be discussed in the maintenance phase below.

There was a general trend of music being less important over time, particularly towards the maintenance phase. When looking at the types of activities being performed, listening to music together seems to be done throughout a relationship, but becomes more common over time. Meanwhile, sharing and discussing music becomes less common; as people get to know each other’s music tastes better, there is less need to talk about it. Making music with a partner peaks during the building phase. Although emotion regulation was not mentioned as often as other codes, there is a consistent trend of music being used more for regulation towards the start of a relationship. Certain trends emerge when looking at which codes are mentioned together, which enables actions to be matched to outcomes. Assessing signals of compatibility with a partner was often mentioned, along with shared music listening, discussing music, and active music making. Meanwhile, people often mentioned increased bonding and connection as an outcome of joint listening and discussing music. Overall, throughout the course of a relationship, music seems to be used less to assess compatibility, and more to deepen relationships.

***Attraction.*** In the attraction phase, music was considered to be of high importance, particularly for intimacy and passion. Participants were most likely to mention listening to music together in public and discussing music in this phase. Previous literature has also suggested that talking about music is a common activity during dating, as it is an easy early conversation to learn about strangers ( Rentfrow & Gosling, 2006). Our participants also frequently mentioned feeling attracted to those with compatible music tastes, which is consistent with assortative mating strategies, that is, that people seek out mates who are like themselves ( Luo, 2017). Simultaneously, many participants also said that they found musical ability to be attractive to a potential partner, which appears to be consistent with the theory that musicality is a sexually selected trait ( Miller, 1999), although individual differences in our sample also show that music is much more important for those who are musical, so it may be mostly musicians who report finding musical ability attractive. Based on the most common outcomes, it seems that participants were assessing potential partners for compatibility through their music preferences. There were also some people who already spoke about a deepening connection with a potential partner during the attraction phase, although this becomes more prevalent in the next phase.

***Building.*** Most participants rated music as equally important during the building phase as in the attraction phase, and again it was highest for the intimacy and passion facets of Sternberg’s (1986) triangular theory of love. Sharing and talking about music as well as shared music listening were just as common as in the attraction phase. However, shared music making also emerged as a frequent activity in this phase for some participants, demonstrating their increased vulnerability with their partners. In these instances, shared music making may have been used as a means of increasing feelings of social closeness, as suggested by previous research ( Dunbar, 2012; Savage *et al*., 2021). Correspondingly, increasing connection with a partner became a much more frequent outcome in this phase; attraction may have already been established, but at this stage people were getting to know each other more. Nevertheless, many participants were still assessing their partners for compatibility, and some also reported noticing incompatibilities more as they got deeper into the relationship. This could reflect the ending of what may be referred to as the ‘honeymoon phase’ or ‘new relationship energy’, which often occurs at the start of a relationship ( Huston *et al*., 1986; Lucas *et al*., 2003), as participants moved towards the maintenance phase of their relationships.

***Maintaining.*** In general, music was seen as less important during the maintenance phase. Contrary to our hypothesis ( Bamford *et al*., 2024), music was not seen as more important for enhancing commitment at this phase, according to self-report ratings. Nevertheless, in the qualitative responses, some participants did mention that music played an important role in their future planning, which was not a feature of prior stages. The most common responses were around choosing music for weddings, for example, “to try and blend our eclectic and different tastes in music into a cohesive couple story” (Participant 49), while others mentioned discussing the songs that they would sing to their future children (participants 33 and 208), or even dreams of “owning a record store together” (Participant 271). It seems that, although participants did not report that music was important for commitment in their relationship on average, there were specific ways in which music was involved in future planning and commitments for some participants.

Other activities mostly decreased in frequency during this phase, except for music listening, which was reported more frequently. Bonding and compatibility were the most common outcomes in this phase, just like in the building phase, although here bonding was more frequent. The way participants discussed compatibility seemed to be different in the maintenance phase, as participants were more likely to talk about how they had overcome differences, or had grown apart, rather than discovering shared musical preferences as they did in earlier phases. As with the previous stages, many participants mentioned that music helped strengthen their bond with their partner, but it also became more common at this stage to begin losing connection, sometimes connected with diverging musical interests.

### Individual differences

Our correlational analysis suggests that music plays a greater role overall and in terms of the three aspects of the Triangular Theory for individuals with higher levels of musical reward and, to a lesser extent, musical expertise. Similarly, individuals with higher expertise and music reward reported talking more about music preferences when they met a potential partner, they tended to place more importance on having similar music tastes and on the partner having high musical ability. The BMRQ further correlated with having an “our song” and seeing music as more important than other leisure areas.

These findings tend to support the idea that music serves as a way to measure similarity with a partner (e.g., Luo, 2017), rather than being a universally attractive trait (e.g., Miller, 2000), which appears to be particularly important for individuals who place a high value on it or have strong musical skills themselves. However, while music appeared to be more relevant to certain individuals, participants at all levels of expertise and musical reward reported musical activities and outcomes in their qualitative responses. Therefore, music has some value for all, for example, in terms of providing insights into other people's personalities, values, arousal preferences, and desire for cognitive stimulation ( Boer *et al*., 2011; Getz *et al*., 2014; Vella & Mills, 2017).

### Limitations and future directions

While the strengths of this study include the mixed-methods design and the large, diverse sample in terms of musical background, some limitations should be noted. Participants were predominantly female and highly educated, which may limit the generalizability of our findings. In terms of measurement, we assessed the role of music in intimacy, passion and commitment using three simple, self-generated questions. As these questions were not based on pre-existing scales, the results should be interpreted with caution. In addition, participants were asked to retrospectively report on their experiences with music in the context of past relationships and relationship stages, which limits causal claims from the co-occurrence analysis, as some responses included multiple experiences or relationships. Consequently, co-occurrence only indicates that actions and outcomes were mentioned together, and not that one caused the other. To overcome this limitation, future research could include recruiting participants who are currently in different phases of a romantic relationship, collecting dyadic data from both romantic partners, or using designs such as experience sampling methods.

 Finally, while this study focused on exploring the role of music in romantic relationships, we did not investigate whether similar results could be achieved by comparing music with other leisure activities for which the couple may have a shared preference, and whether these findings could be extended to other types of relationships, such as close, non-romantic friendships. Existing research suggests that discussing musical preferences is the most frequent topic when initiating a relationship with a stranger ( Rentfrow & Gosling, 2006). While the present findings extend this by showing that sharing and discussing musical preferences is the second most frequently mentioned activity in romantic relationships, the question remains: is musical compatibility equally important for romantic and non-romantic relationships?

## Conclusion

Considering the relevance of music for social bonding as well as the vast number of songs that address love, it is astonishing how little attention has been paid to study the role of music in romantic relationships. The current study provides a first systematic investigation of the relevance of music for the three core aspects of love across the three major phases of romantic relationships. Based on the results the core affordance of music appears to relate to intimacy: learning about, getting closer, and feeling more connected to a partner. The capacity of music to facilitate social intimacy resonates well with the access-awareness-agency model of music-based social-emotional competence ( Saarikallio, 2019) and the broad literature on music as social-bonding in general (e.g., Savage *et al*., 2021; Tarr *et al*., 2014). This raises the important question of whether the role and purpose of music in romantic love truly differs from the role that music serves for human social behaviour in general. Activities such as music listening during sex, music that inspires future plans for family, or the use of music to ignite passion are perhaps special for romantic relationships, but testing the broader similarities with other types of relationships could be an interesting avenue for future research. Overall, this study offers important new insights into how music is present in romantic relationships, which can be meaningful from music research to couple therapies and broader approaches of studying human relationships.

## Ethical approval and consent

This study was considered exempt from ethics committee approval in accordance with the guidelines of the local university ethics committee. Prior to participation, the participants were fully informed about the study and provided informed consent in accordance with the principles of the Declaration of Helsinki. Participants gave their written consent to the following statement by clicking on a on the 'I agree' button:

*I am at least 18 years old and freely agree to participate in this research study. I have been informed about the purpose, procedures, and nature of the study and understand them fully. I am aware that my participation is voluntary, and I have the right to withdraw from the study at any time without any penalty or consequences. I understand that the data collected during the study will be kept strictly confidential and anonymized, ensuring my privacy. I hereby grant permission for the data generated from my participation to be used in the researcher's publications on the topic of the study.*

Data were securely stored on the university's server to ensure the anonymity and confidentiality of all participants.

## Data Availability

The dataset and analysis code for this study are available in the following OSF repository: Vigl, J., Bamford, J. S., Saarikallio, S., & Fleckenstein, A. M. (2024, November 22). Music Across the Love Span. https://doi.org/10.17605/OSF.IO/EH8WN This projects contains the following underlying data: Extended data: **extended_data.docx**: This document contains five additional tables (Tables S1–S5) and one figure (Figure S1). **co-occurenceMatrix_full.xlsx**: Full co-occurrence matrix of action and outcome codes overall and separately for the relationship phases. Quantitative data: **data_wide.csv**: Anonymized answers to the quantitative questions in wide format. **data_long.csv**: Anonymized answers to the quantitative questions in long format. Codebooks for both datasets are provided in the same location Qualitative data: **qualitativeData_wide.csv**: First-order codes assigned by two coders in wide format. The ID column in this dataset refers to the participant index number that is also provided in the quantitative datasets **qualitativeData_long.csv**: First-order codes assigned by two coders in long format. The responseID column in this dataset refers to the participant index number that is also provided in the quantitative datasets **openResponses.xlsx:** This file contains the qualitative data (responses to the open-ended questions) in a de-identified format (i.e., without the participant ID), together with the phase for which the response was given, as well as the first-order codes and second-order themes assigned by both coders. Data analysis: **dataAnalysis.R**: All quantitative and qualitative analysis performed for this article. Data are available under the terms of the Creative Commons Zero “No rights reserved” data waiver (CC0 1.0 Public domain dedication).
