# Supplementary material for: Music across the love-span: a mixed methods study into the use of music in romantic relationships
Source: Open Res Eur. 2025 Oct 31;4:275. Originally published 2024 Dec 24. [Version 2] doi: 10.12688/openreseurope.19016.2 (PMC12631075; doi:10.12688/openreseurope.19016.2)
Supplement: Peer review history

# Reviewer report: Reviewer response for version 2

**DOI:** 10.21956/openreseurope.23478.r63297

**Published:** 2025-11-22

**Authors:** Adam Bode

**Affiliations:** 1 The Australian National University, Canberra, ACT, Australia

Thankyou for the opportunity to review the manuscript Music across the love-span: a mixed methods study into the use of music in romantic relationships.

 It investigates, using quantitative and qualitative methods, the perceived importance of music on three components of love across different stages of a romantic relationship. I generally think the article is interesting and should be indexed with some changes. I have read the comments of reviewer 3 and agree with their assessment. I will not comment on issues they have already commented on. I also have no expertise in qualitative analysis, so cannot assess that aspect of the manuscript.

**Introduction:**

 The authors misuse the term “romantic love”. Romantic love in the English language (Collins, 2025), according to the APA (American Psychological Association, 2018), according to Sternberg’s theory (Sternberg, 1986), and according to the convention among love researchers (Bode & Kowal, 2023) is a motivational state that commonly occur in the early stages of a romantic relationship, is associated with extremely strong cognitions, emotions, and behaviours, and usually lasts in the vicinity of 18 months (Bode & Kushnick, 2021). What you mean is “love in romantic relationships”, which encompasses both romantic love and companionate love (the less intense love that often exists in long-term married couples - (Walster & Walster, 1978).

 Please change your references to “romantic love” with the term “love in romantic relationships”. You should probably also define what is meant by this term, and specify it includes both romantic and companionate love. There are others, including one notable love researcher, who misuses this term, so it is understandable that you would also misuse the term. Please fix.

 When you talk about the validations of Sternberg’s theory, you should probably also include (Kowal et al., 2024) which validated a short-version of the scale in heaps of languages.

 Your romantic relationships stages subsection relies heavily on work that is rather old. Please emphasise that this is just one framework and provide evidence in support of it from the past ten years if this available. It’s fine to use this framework as a heuristic, but please be aware that the timeframes provided are nonsense, and there is substantial variation in the trajectory of romantic relationships. This framework also does not recognise that the norm among romantic relationships is to be friends first, so a type of bond is usually formed in advance of the romantic relationship. It also does not consider different types of love.  Please emphasis that this is a “useful” framework for the current purpose.

 Please define mate choice.

**Methods**

 You conflate sex and gender. Female and male are sexes. Non-binary is a gender identity.

 If possible, please check to see if there is an effect of age on the results of the quantitative analysis. Music serves different functions across the lifespan. And for people who are not musicians, the importance of music peaks in adolescence and early adulthood and becomes less important with age.

If applicable, is the statistical analysis and its interpretation appropriate?

I cannot comment. A qualified statistician is required.

Is the study design appropriate and is the work technically sound?

Yes

Is the work clearly and accurately presented and does it engage with the current literature?

Yes

Are the conclusions drawn adequately supported by the results?

Yes

Are sufficient details of methods and analysis provided to allow replication by others?

Yes

Are all the source data and materials underlying the results available?

Yes

Reviewer Expertise:

Love

I confirm that I have read this submission and believe that I have an appropriate level of expertise to confirm that it is of an acceptable scientific standard, however I have significant reservations, as outlined above.

## References

[1] American Psychological Association. (2018). APA Dictionary of Psychology - Romantic Love. APA. Retrieved 30 September from https://dictionary.apa.org/romantic-love.

[2] : Toward consistent reporting of sample characteristics in studies investigating the biological mechanisms of romantic love. *Frontiers in Psychology* .2023;14: 10.3389/fpsyg.2023.983419 10.3389/fpsyg.2023.983419 PMC1019291037213378

[3] : Proximate and Ultimate Perspectives on Romantic Love. *Frontiers in Psychology* .2021;12: 10.3389/fpsyg.2021.573123 10.3389/fpsyg.2021.573123 PMC807486033912094

[4] Collins. (2025). Defintion of "romantic love". Collins. Retrieved 30 September from https://www.collinsdictionary.com/dictionary/english/romantic-love.

[5] : Validation of the Short Version (TLS-15) of the Triangular Love Scale (TLS-45) across 37 Languages. *Archives of Sexual Behavior* .2024;53(2) : 10.1007/s10508-023-02702-7 839-857 10.1007/s10508-023-02702-7 PMC1084434037884798

[6] : A triangular theory of love. *Psychological Review* .1986;93(2) : 10.1037/0033-295X.93.2.119 119-135 10.1037/0033-295X.93.2.119

[7] Walster, E., & Walster, G. W. (1978). A new look at love. Addison-Wesley.

# Reviewer report: Reviewer response for version 2

**DOI:** 10.21956/openreseurope.23478.r63211

**Published:** 2025-11-19

**Authors:** Andrea McGraw Hunt

**Affiliations:** 1 Rowan University, Glassboro, New Jersey, USA

Thank you to the authors for these revisions, I appreciate their consideration of my review and responses. I have no additional comments to make on this study and look forward to its indexed.

If applicable, is the statistical analysis and its interpretation appropriate?

I cannot comment. A qualified statistician is required.

Is the study design appropriate and is the work technically sound?

Partly

Is the work clearly and accurately presented and does it engage with the current literature?

Yes

Are the conclusions drawn adequately supported by the results?

Yes

Are sufficient details of methods and analysis provided to allow replication by others?

Partly

Are all the source data and materials underlying the results available?

Yes

Reviewer Expertise:

I am a board-certified music therapist with expertise in a wide range of qualitative and quantitative methodologies, particularly neuroscience and chronic pain. I have supervised graduate research involving survey design.

I confirm that I have read this submission and believe that I have an appropriate level of expertise to confirm that it is of an acceptable scientific standard.

# Reviewer report: Reviewer response for version 2

**DOI:** 10.21956/openreseurope.23478.r63299

**Published:** 2025-11-07

**Authors:** Claire Howlin

**Affiliations:** 1 Trinity College Dublin, Dublin, Ireland

Many thanks for inviting me to review this interesting paper, which aims to explore how the importance that you assign to music is related to romantic relationships, musical expertise and musical reward. The authors find that the importance that you assign to music in a romantic relationship is related to how much reward you derive from musical experiences. Based on small to medium correlations. The authors identify that the perceived importance of music is different for each aspect of love, intimacy, passion and commitment, but unfortunately the nature of these differences are not clearly reported in the results section. The authors also identify that the perceived importance of love is different between the maintenance phase and the building and attraction phase, but it is not clear at which phase that music plays a greater role. Similarly the discussion section, does not accurately reflect the results that are reported. Clearer and more consistent reporting throughout the manuscript is needed before this study could be indexed.

 Please see details below of issues that need to be addressed.

 Introduction:

 At the end of this section an aim is put forward: to explore perceptions of the role of music at different stages of romantic relationships and to examine which musical activities and their outcomes influence partner choice and facilitate or inhibit romantic feelings.

 Were musical activities examined as part of this research?

 Which outcomes were examined as influencing partner choice?

 Did the study examine how musical activities inhibit romantic feelings?

 What do the authors mean by ‘role of music’?

 Check the text thoroughly for minor typos e.g. ‘This combination’ under present study

*RQ1*: How important is music in the context of romantic relationships, and does it contribute to strengthening intimacy, passion, and commitment at different stages of romantic relationships?

 Result: The importance that you assign to music in a romantic relationship is related to how much reward you derive from musical experiences. Based on small to medium correlations

 Research question 1 is set up as causational, but the analysis is correlational. Based on the data and analysis it is not possible to ascertain if the importance a person assigns to music strengthens intimacy.

 Were the hypotheses registered before the data collection, or is this an exploratory study? In the results section RQ2 is framed as an exploratory analysis, so it might be better to state that more explicitly here.

 RQ2: The first hypothesis is not clear four variables are listed (musical expertise, sensitivity to musical reward, perceived importance of music in romantic relationships and perceived importance of music in different aspects of love), and the direction of the correlation is not stated. Does this need to be simplified, or separated into additional hypotheses?

 “musical expertise and sensitivity to music-related reward may correlate with the perceived importance of music in romantic relationships overall, as well as in different aspects of love."

 There is a large drop off between the number of participants who started the survey (402) , and the number included in the study (174). Could you provide more details on how the final sample was determined, and any decisions made on data missingness?

 Limitation:

 The qualitative aspect of the study was limited to a text box response, without any follow-up questions or prompts to elicit further detail from participants. This would likely limit the depth of information shared by participants.

 Participants agreed that music has different levels of importance for different aspects of love and phase. However the effect size for phase is extremely small np2 = 0.07. The extremely small effect size needs to be clarified throughout the results and discussion. Indeed many authors may decide to report such a small effect as almost negligible. 

 For RQ1 the quantitative results report that there are significant differences between all aspects of love, but it does not report the direction or magnitude of the differences. Please report the direction of the differences.

 The following text appears to first say that there is no difference in the reported general role of music across the three phases of a relationship, but then the means are reported as if there is a difference

 “As shown in Figure 1, participants reported a high general role of music in all three phases ( *M* = 4.00, *SD* = 0.89). The importance of music for the three components was higher in the attraction ( *M* = 3.61, *SD* = 1.08) and building ( *M* = 3.59, *SD* = 1.02) phases than in the maintenance phase ( *M* = 3.31, *SD* = 1.05), while it appeared to be most important for intimacy ( *M* = 3.96, *SD* = 0.97), followed by passion ( *M* = 3.76, *SD* = 0.99), and commitment ( *M* = 2.79, *SD* = 1.18).”

 Table 2. Musical expertise variable appears to have a Mean of 0.00 (SD = 0.83). How could the Mean be zero when the lowest score on the Ollen scale is 1, and 43% of the sample reported a score of 2-5?  

 It’s unusual to see a frequentist analysis applied to a qualitative theme, its unclear what the purpose of this is, or how to interpret it.

 There doesn’t appear to be any qualitative descriptions of the themes themselves, and this needs to be provided.  

 Discussion:

 The opening lines of the discussion do not strongly convey the main findings reported in the results. Consider revising to make this stronger/clearer.

 In the results section a key finding is that the importance that you assign to music in a romantic relationship is related to how much reward you derive from musical experiences. Based on small to medium correlations. But this is not clearly reflected in the discussion.

 The discussion states that the results demonstrate a medium to high overall importance of music. Where is this demonstrated? Do you want to report the descriptive statistics for this variable.

 The discussion states that the perceived importance of music is stronger for intimacy and passion than for commitment, but this is not demonstrated in the results. Although differences are reported, the size and direction of the differences is missing. “Pairwise comparisons using Bonferroni correction indicated significant differences between all relationship aspects ( *p* < .001). Regarding relationship phases, there were no differences between the attraction and building phases ( *p* = 1.00), but there were differences between the maintenance phase and both the attraction and building phases ( *ps* < .001). “

 Please report the main effects in full in the results section.

 The discussion states that musical expertise is associated with higher ratings for the overall role of music and its contribution to the three aspects of love. However, the relationship between musical expertise and the overall role of music is .22 indicating an extremely weak correlation, and no significant correlation is found between musical expertise and three aspects of love. Please re-phrase the discussion section so that it accurately describes the results section.

 Would you consider presenting the relationships between BMRQ and aspects of love first, and then in a new sentence present the relationships between musical expertise and aspects of love. Seperating them will make it easier to speak more precisely.  

 Limitations and strengths.

 One of the strengths put forward is that there is a rich musical background, however the mean level of musical expertise reported is zero in Table 2. Please examine the reporting of the data and revise statements if necessary.

If applicable, is the statistical analysis and its interpretation appropriate?

Partly

Is the study design appropriate and is the work technically sound?

Yes

Is the work clearly and accurately presented and does it engage with the current literature?

Yes

Are the conclusions drawn adequately supported by the results?

Partly

Are sufficient details of methods and analysis provided to allow replication by others?

Partly

Are all the source data and materials underlying the results available?

Yes

Reviewer Expertise:

Music Cognition, Health Psychology

I confirm that I have read this submission and believe that I have an appropriate level of expertise to confirm that it is of an acceptable scientific standard, however I have significant reservations, as outlined above.

# Reviewer report: Reviewer response for version 1

**DOI:** 10.21956/openreseurope.20577.r59623

**Published:** 2025-09-26

**Authors:** Daniel L Bowling

**Affiliations:** 1 Stanford University, Stanford, California, USA

OVERALL IMPRESSION AND RECOMMENDATION

 It was a pleasure to read this informative and clearly communicated analysis of music in the context of normative human pair bonding. The length of my review reflects my thorough reading of the text and suggestions to improve it, rather than an abundance of problems with the paper. That said, I have several more substantive criticisms having to do with limitations/interpretation and data presentation/analysis. These are presented under “GENERAL CRITIQUES” or with *** *** in “SPECIFIC COMMENTS”. Provided that the authors can address these criticisms or better explain their perspective, I would happily recommend this manuscript publication. This is important but neglected topic and I applaud the author’s for getting the ball rolling. My recommendation is minor revision.

 GENERAL CRITIQUES:

 (1) The fact that the sample is nearly 6:1 female:male needs to be mentioned up front. One might very well expect the role of music in courtship / mating / bonding to be different women and men. Likewise, age would seem to be an important predictor, given that both mating behavior and engagement vary as a function of age. More broadly, I recommend that the author’s consider the limitations of their sample more seriously. E.g., given that the data was collected primarily from highly musical young women, how well should we expect them to generalize, e.g., to older couples, to men, or to those who haven’t spent 15 years playing an instrument (on average).

 Culture also relevant here. Understanding the absence of primary research, any data (or even anecdotes) about music and romance from other cultures, would provide useful context.

 (2) The degree to which the overall high importance of music in romantic relationships reported here was related to individual differences in sensitivity to musical reward remains unclear. Why not approach this question empirically? For example, by splitting the data by high vs. low sensitivity to musical reward, and then comparing ratings.

 (3) The positive/negative component of the data is really interesting (i.e., whether music promoted or obstructed romance), but doesn’t appear to have been systematically analyzed or discussed despite being present in the codes. Can the author’s provide any clarity here? E.g., A clearer indication of negative/positive frequency, and what this might, or might not imply? Perhaps the nature of the study is biased towards collecting positive accounts, but even so, people choosing to describe instances of incompatibility is interesting. How rare was it?

 SPECIFIC COMMENTS ON THE TEXT, BY SECTION:

 Most of these are aimed at improving the text or clarify. Those in bold are more important, and/or harken back to the above.

 ABSTRACT:

 “174 participants”

 -Include male/female breakdown

 INTRODUCTION:

 “These effects are often attributed to the synchronisation involved, as engaging in synchronised actions commonly leads to increased feelings of social affiliation, trust, and prosocial behaviour”

 -More clearly define “synchrony”, which is a polysemous term. E.g., “These effects are often attributed to synchrony, as interpersonal temporal coordination of action commonly leads to …”

 “Social space that aligns the intentions of participants”

 -intentions and emotions?

 “Little work…commitment in romantic relationships”

 -Add biological context for why this is interesting? (this = “how music might influence partner choice or enhance feelings of intimacy, passion or commitment in romantic relationships”. For example, in nature, singing is frequently associated with pair bonding (e.g., in gibbons and birds), leading to theories that center sexual selection in the evolutionary origins of music.

 “One couple-specific”

 -Maybe: “A prominent example of music in romance is the “couple-defining song”.

 “According to Harris et al. (2020) …” Nice to have a number here, but also wanting more context on the nature of the study. Something like, “Accordingly to Harris et al.’s analysis of XX couples in the US...”

 In the next paragraph: “Other research has focused on …”

 -Switch the order of the examples given to add focus to the more relevant point? I.e., research has found that musical traits are attractive, as is creativity more generally.

 “That musicality”

 -This term needs unpacking or qualification. In this context, it might be better to say “musical ability” if that’s what’s meant, or “musical interest”. -If “musicality” is preferred, briefly define it, e.g., “musicality (i.e., musical interest and/or ability)”

 “Harwood & Wallace (2021)”

 -Is there anything about the musical experience or ability of the couples in this paper that might represent a potential confound? For example, were these couples more compatible because both partners were musical? What is the relevant conclusion that the readers should take from this?

 “RQ2... personal characteristics”

 -Clarify the kind of personal characteristics that are being referred to here.

 ***

 “RQ2… so musicality may correlate with the perceived importance of music”

 -Clarify meaning. Is it something like “so higher musicality in a couple may indicate that both partners perceive music to be important”? Not clear what this has to do with other music-associated personal characteristics. Further, I’m not sure what adding romantic context to the basic question of what personal characteristics are associated with music brings to the table. Seems like I’m missing something here about what the authors are trying to get at.”

 ***

 “While RQ1 and RQ2 will be answered with quantitative data”

 -“answered” should be “addressed”

 METHODS:

 Specify education level of remaining 10% (e.g., “10% less than high school”). Is “highly educated” the best characterization? Roughly a third only finished high school, while 10% had less than that.

 “Regarding musical background”

 -Add generalization at the top of this paragraph to help the reader summarize the musical sensitivity of the group? It’s not easy to square the fact that 61% played/sang for 16 years on average with 51% reporting being non-musicians. These are imperfect measures but the reader still wants to know how they should think about this aspect of the sample… Highly musical, not very musical, etc…. “Diverse” (from the discussion)? Make the case.

 “Participants were explained”

 -Maybe: “Participants read text written by the experimenters explaining…”

 “Music-related reward experience”

 -“sensitivity to musical reward” (what the BMRQ measures)

 “Quantitative measures”

 -Presenting the questions in this paragraph in table form? Currently hard to parse and map to methods.

 “continuous composite score...(alpha = 0.86)”

 -Specify “Cronbach’s alpha”

 -Move this stat to after “as these four items were internally consistent”.

 -Restate what “these four items were” in parentheses after “four items”?

 “music-related reward”

 -Better: “sensitivity to musical reward” ?

 “The exact wording…” Move this reference to the OSF repository to the start of the survey description. This way the reader knows right away that they can go look at the survey directly if they need to, so don’t need to get caught up on trying to parse exactly what it was from the methods alone.

 Was the survey done in German? If so, it is worth mentioning, that the “wordings” provided have been translated for this manuscript.

 “Data Analysis”

 “Greenhouse-Geisser corrections as necessary”

 -Add “for violations of sphericity”

 -Were tests done to determine if GG correction was necessary? Describe.

 -Describe stats software too.

 “The first analysis predicted the role of music…the overall importance of music”

 -In describing these models, it would be useful to know specifically what questions pertain to these variables.

 “Associations between personal characteristics…”

 -As noted above, the meaning of personal characteristics should be clarified, especially with respect to which survey questions were considered to assess it.

 “Pearson correlations were used to identify…”

 -Given that so many correlations have been tested, a correction for multiple comparisons seems appropriate, e.g., Benjamini-Hochberg.

 RESULTS:

**Terms like “perceived general role of music” are difficult to map to specific originating questions, especially on the first read through before the reader understands the results. A table listing the quantitative questions and codes, like Q1, Q2, etc. would help**

 “To determine whether there are personal characteristics associated with the role of music in romantic relationships”

 -There almost certainly are, right? But the measures of “personal characteristics” included here are fairly limited…

**“…with the assessed personal characteristics: demographics…”**

**-Here we finally get a clear definition of what “personal characteristics” means in the context of this paper. This explanation should be provided earlier, ideally with some logic for why these have been selected.**

 Are Pearson correlations appropriately applied to a variable like gender or education, which have presumably been treated categorically? Might want to explain the numerical coding scheme.

 Figure 1. It’s a bit confusing to have box plots and violin plots on top of each other (the meaning of the gray circles is not described in the legend). Recommend adding the actual data points and choosing either violins or boxes

 ***

 Table 1. As shown, in addition to correlations been demographics and outcomes, correlations are also being assessed between outcomes and outcomes, and demographics and demographics. The later two cases are not of any importance to RQ2. Given the multiple comparison problem, I recommend only testing the correlations the authors care to test. For clarity, I also recommend separating the “personal characteristics” and outcomes by placing them on different axes.

 ***

 “…achieved at least fair agreement”.

 -Which codes had the lowest agreement?

 ***

 Under “Aggregate dimensions:..” How are percentages of responses related to percent of respondents? Do I need to worry that a subset of respondents have biases the results?

 ***

 “(re)connection” and “(co)regulation”.

 -Clarify whether these terms imply, e.g., “connection and reconnection” or only reconnection.

 “accounting for 68.6%, 73.0%, and 75.7%”

 -add “of responses”, presuming that is correct

 “…signaling as an outcome, indicating attraction or compatibility”

 -Add an example in parentheses here? To explain directly how music was used to signal attraction or compatibility

 “…was represented in 7.6% of the codes”

 -Does this mean that 7.6% of all codes were 3A? If so, better to say directly, and include the letter code.

 “…only one code appeared in nearly five percent of the responses...”

 -This cutoff seems arbitrary, even considering that it is only for descriptive purposes. Maybe something like: “In the outcome dimensions, codes were more variable, with the most frequent only occurring in 4-5% of responses. Examples include…”

 “(in)compatibility”

 -Explain. Were responses mostly referring to compatibility? What proportion were about incompatibility? Does musical incompatibility predict leaving a relationship?

 ***

 In the RESULTS in general, it would be great to have more letter codes in the text to help the reader map to the figure /table. Currently, this is done sometimes but not consistently.

 ***

 When summaries of music uses are introduced, e.g., “incorporating music into future plans” or “coping with differences”, include paraphrased example in parentheses (e.g., “planning music for a wedding”). This is done sometimes but not others.

 “A more detailed heart of”

 -typo, missing word “map”

 ***

 Figure S1 - this seems like the most primary representation of the data? Is it really too busy for the main text, e.g., in place of Figure 3? It would allow the reader to see a complete code-based summary of the data in one picture. If Figure 3 is left as is, specify what the numbers that appear on the figure signify in the legend (are these as important to show as the coded data?)

 ***

 Figure 4 legend.

 -Delete “note”. This information is key to interpreting the figure. “How often” in terms of absolute number or percentage? Please specify

 DISCUSSION:

 “…provides an overview of how important music is at different relationship stages, by enhancing different facets of love”

 -Do the data really show this? The importance data (figure 1) don’t seem to differentiate the different facets of love very well at all, other than commitment, which may have more to do with the age of the sample. Also, the case for enhancement (causal) as opposed to association, or even detriment, should be more clearly made.

 ***

 “With stronger correlations present in individual with higher levels of musical expertise and music reward experience”

 -Seems inaccurate to equate musical expertise and musical reward sensitivity? The latter appears much more strongly related (~2x)

 ***

 “…with listening to music, sharing or discussing music, and making music together as the most common”

 -Quantify. E.g., “accounting for XX% of all action codes”, or list relative proportions

 “Outcomes were less frequency mentioned,”

 -Quantify: percentage?

 “Even though we have generated the codes purely data-driven, some parallels with Sternberg’s Triangular Theory can be drawn”

 -Considering that Sternberg’s Theory is key to the theoretical framework in which this work originated, is it really surprising that some parallels are found? Seems not, especially given that data coding was guided by the researchers. I don’t take issue with the methodology, so much as this rhetoric, which doesn’t seem to reflect the facts at hand.

 “Our participants also frequently mentioned feeling attracted to those with compatible music tastes”.

 -Quantify. How common? List percentage, or at least point to figure where the reader can quickly gauge empirical support.

 ***

 “Simultaneously, many participants... “

 -Quantify or point. I understand that the author’s may not want to stick a bunch of numbers in the discussion. But the numbers / proportions need to be made more prominent somehow. Adding them to the text seems fine to me but a table will also work.

 ***

 “… so it may be mostly musicians who report finding musical-ability attractive”

 -Don’t the author’s have the data to address this empirically?

 ***

 “Individual differences… BMRQ”

 -This section is stands out of the lack of associated data (unless I missed it!)

 -How prominent were the differences between people with high and low sensitivity to musical reward?

 ***

 “Based on the most common outcomes … “

 -List examples, percentages, etc. help the reader understand that what you’re saying is accurate / representative.

 “However, shared music making also emerged as a frequent activity in this phase for some participants”

 -Nice qualifying point. How frequent?

 ***

 “Contrary to our hypothesis, music was not seen as more important for enhancing commitment at this phase, according to self-report ratings”…

 -Why is self-report a problem here but not for the rest of the author’s data? Why doubt this result and not others… This speaks to deeper methodological concerns, e.g., “using music to enhance commitment” isn’t something I’d necessarily report either, despite the fact that my partner and I always listen to music when we’re hanging out (25 years in). Those musically-supported hangouts definitely bind us together, though we are not explicitly using the music to enhance commitment. Maybe the questions just aren’t right.

 ***

 LIMITATIONS

 “...strengths of this study include mixed-methods design...”

 -The value of qualitative data will not be obvious to many readers and should probably be explained rather than assumed (if not here, then above - sorry if I missed it.

 “…large, diverse sample in terms of musical background”

 -Sample not all that large for an online survey study. Also, not clear to me that the sample stands out for being diverse in musical skills. Seems like they were a very musical group (15 years playing music/singing on average?)

 “predominantly female”

 -An understatement.

 “highly educated”

 -Do the author’s expect that this factors was relevant? Was it relevant in their data? Better to provide insight instead of simply calling this a limitation…. Or at least explain why examining these factors more seriously here doesn’t make sense (e.g., not enough males to compare with females).

 CONCLUSION

 “access-awareness-agency” model… strange to bring this up here having not even mentioned or described it elsewhere in the paper. Would love to see it introduced earlier and explained if it is important to contextualizing this work.

If applicable, is the statistical analysis and its interpretation appropriate?

Partly

Is the study design appropriate and is the work technically sound?

Yes

Is the work clearly and accurately presented and does it engage with the current literature?

Yes

Are the conclusions drawn adequately supported by the results?

Partly

Are sufficient details of methods and analysis provided to allow replication by others?

Yes

Are all the source data and materials underlying the results available?

Yes

Reviewer Expertise:

Biology of music; music & health

I confirm that I have read this submission and believe that I have an appropriate level of expertise to confirm that it is of an acceptable scientific standard, however I have significant reservations, as outlined above.

# Response

**Published:** 2025-10-22

**Authors:** Julia Vigl

**Affiliations:** Psychology, Universitat Innsbruck Fakultat fur Psychologie und Sportwissenschaft, Innsbruck, Tyrol, Austria

Dear Dr Bowling, 

 Thank you very much for taking the time to read our article and for providing such helpful and detailed comments, all of which we have tried to address in our revisions. We have submitted a revised version of the manuscript, which should be available online soon. Below, we summarise the issues you raised and explain how we addressed each comment, alongside the corresponding changes to the manuscript.

**Responses to general critiques: **

1. Thank you for highlighting the importance of **acknowledging the unequal gender distribution and limitations in generalizability** of our data. To address this issue, we included the percentage of female, male, and non-binary participants in the abstract, mention the sample composition at the beginning of the discussion, and expanded the 'Limitations and Future Directions' section. There, we also discuss how the results might differ with a more balanced sample composition. Regarding musical background, we would like to clarify that the average of 15 years of instrument playing refers to the 61% of participants who indicated that they play an instrument or sing, not to the total sample. In the total sample, 57% considered themselves non-musicians. 
2. We appreciate the suggestion to **empirically examine the role of musical reward sensitivity in more detail**. In the revised manuscript, we have included this analysis immediately following Table 2. Specifically, we reran the ANOVA used for RQ1, adding BMRQ group (low vs. high, based on a median split) as a between-subjects factor. The results revealed a significant main effect of BMRQ, indicating that participants with higher music-related reward sensitivity reported a greater overall role of music and stronger contributions of music to the three aspects of love. No significant interactions were observed, showing that the relative pattern across love aspects and phases did not differ by BMRQ group. We have also added a figure to the OSF repository that shows boxplots for the importance of music overall and in strengthening the three aspects of love separately for the low and high BMRQ groups.
3. Thank you for highlighting the importance of **considering negative experiences of music in romantic relationships**. we have added a dedicated subsection entitled 'Negative experiences' to the qualitative results, placed after the analysis of first-order codes. This section details the frequency of negative codes overall and by phase, and identifies the most prevalent negative codes. We moved the illustrative participant quote from the first-order code description to this new subsection.

**Responses to specific comments on different manuscript sections** **Specific comments on the abstract and introduction: **We have incorporated your suggestions as follows:

- In the abstract, we have added the number of male and female participants.
- In the introduction, we clarified key concepts, such as synchrony, and explained why studying musical experiences in couples is particularly relevant from an evolutionary perspective. We also provided more information about the samples used in the cited studies (Harris et al., 2020; Harwood & Wallace, 2021). Furthermore, we rearranged two paragraphs to improve the logical flow, moving the studies on music and attraction before the study on couple-defining songs.
- Section “the present study”: We expanded the description of RQ2 to specify that we focused on musical background and sensitivity to musical reward when exploring the moderating role of individual differences. Correlations with demographic variables were exploratory and are therefore not emphasized here (please see our next answer for more details). 

**Specific comment regarding RQ2 (individual differences): **We apologise for the imprecise wording of research question 2. This research question aimed to examine whether the perceived importance of music for romantic relationships, both in general and with regard to specific aspects of romantic love such as intimacy, passion and commitment, varies across individuals, particularly in relation to musical background and sensitivity to musical reward. Investigating these associations is important for interpreting and contextualising our findings, specifically whether the role and potential benefits of music in romantic relationships are generally experienced by all individuals, or whether they are primarily experienced by those with greater musical expertise or appreciation of music. We tried to phrase this better in the section “the present study”. 

**Specific comments on the methods: **Thank you for your suggestions on how to improve the Method section. We have incorporated all of them, as detailed below:

- Sample description: In the new version of the manuscript, we provide more details on the educational levels of the participants, and have clarified the overall level of musical background. In particular, we have added a statement noting the musical diversity of our sample, reordered the presentation (first noting that 57% of participants identified as non-musicians, followed by details on those who play or sing), and included further information on the distribution of individuals playing an instrument among amateur and (semi-)professional musicians. 
- Measures: We added a table (now labelled Table 1 and located in the method section) showing the exact wording of the self-generated questions regarding the general role of music and its perceived contribution to intimacy, passion and commitment, alongside response labels. We also state that the survey could be completed in either German or English, with English versions of the questions available on OSF and German versions available upon request.
- Data analysis: We specify that we tested the sphericity assumption using Mauchly's test and applied Greenhouse–Geisser corrections where necessary. We also note that all analyses were conducted in RStudio and that we meant Cronbach's Alpha when mentioning “alpha” for internal consistency. 
- Correlations: Following your comment regarding the correction of p-values, we have revised the correlation analyses. Table 2 (formerly Table 1) now only reports variables relevant to RQ2 and all p-values have been corrected using the Benjamini–Hochberg method. The same was applied to the tables in OSF. 
- Finally, we have carefully revised the wording of the quantitative measures section in line with your suggestions.

**Specific comments on the quantitative results: **Thank you for your helpful suggestions on how to improve the analysis and presentation of our quantitative results. In response, we have made the following changes:

- Measures: We added a table (now labelled Table 1 and located in the method section) showing the exact wording of the self-generated questions regarding the general role of music and its perceived contribution to intimacy, passion and commitment, alongside response labels.
- We have rephrased the way in which we introduce the examination of individual differences, clarifying that we would like to explore whether the role of music in romantic relationships is moderated by music-related characteristics, namely musical expertise and music-related reward sensitivity. 
- We simplified Figure 1 by presenting only boxplots (rather than combining boxplots and violin plots). We also revised the figure note to explain the meaning of the jittered points and the black circles.
- Correlations (now Table 2): We revised the correlation table to include only the variables relevant to RQ2, showing correlations between the role of music and personal characteristics rather than providing a full correlation matrix. All p-values were corrected using the Benjamini–Hochberg method, yielding largely unchanged results. We now also use Spearman-correlations for all correlation tables in the manuscript and extended data. The same correction has been applied to Tables S1 and S2 in the extended data file on OSF.

**Specific comments on the qualitative results: **We really appreciate your feedback on the qualitative results, which helped us make substantial improvements. In response to your comments, we made the following changes: 

- Presentation of code/theme/dimension frequency: Thank you for raising the question about how the percentages were calculated. In the original version, we reported percentages based on the proportion of all identified codes. Importantly, the results were not biased by single participants providing longer or more detailed responses, as each code was assigned only once per response, even if mentioned multiple times. Upon reflection, however, we agree that when presenting second- and third-order themes and dimensions, it is more meaningful to report the percentage of participants who mentioned each theme or dimension than the percentage of codes. This approach avoids potential inflation for themes with many similar codes and provides a clearer, participant-focused representation of the data. Accordingly, we revised the manuscript to consistently report the percentage of participants who mentioned each code, theme, or dimension. In the data analysis section, we clarified that each code was counted only once per participant per response. The overall pattern of code/theme/dimension prevalence remained largely unchanged.
- Other revisions to the qualitative results: 
  - We corrected the typo noted in the review
  - We specified the two codes with the lowest inter-coder agreement.
  - We incorporated the suggested rephrasing, clarifying the concepts of connection and reconnection within the bonding dimension, adding examples to the aggregate dimension descriptions, and expanding on codes that might not be immediately clear.
  - We moved the former supplementary Figure S1 into the manuscript (now Figure 4), which now shows single-code prevalence across relationship phases.
  - We revised the description of the previous Figure 4 (now Figure 5) for greater clarity.
  - We ensured consistent inclusion of code/theme names throughout the results section.

**Specific comments on the discussion, limitation, and conclusion: **Thank you for your helpful comments on the discussion. We tried to incorporate all your suggestions, resulting in the following changes: 

- We have revised the beginning of the subsection “Music across the lovespan” to avoid overstating our findings. The revised text clarifies that our study examined participants’ perceptions of the role of music, rather than demonstrating causal effects. We now emphasize that commitment was the most clearly differentiated facet and that intimacy and passion received similar ratings. 
- We also clarified that the correlations between the perceived roles of music were stronger for sensitivity to musical reward than for musical expertise.
- In response to your suggestion to include absolute/relative frequencies, we added percentages to the first-order codes mentioned in the discussion. However, we refrained from adding percentages to the nine second-order themes and aggregate dimensions because these are already reported in the Results section.
- We worked to better integrate the qualitative and quantitative findings. For instance, we now discuss that some participants mentioned the importance of a partner’s musical ability in the qualitative data, while the quantitative data revealed that this was more relevant for individuals with stronger musical backgrounds and greater sensitivity to musical reward.
- One comment mentioned that the section on individual differences and the BMRQ would stand out because it is not associated with any data. We would like to clarify that this section summarizes the correlational analyses conducted for Research Question 2, in which we examined the associations between musical background, sensitivity to music-related reward, and the perceived role of music in strengthening the three aspects of love across relationship phases. In response to a comment regarding the results, we added an ANOVA with BMRQ as a between-subjects factor, and we now mention this result in the discussion section as well.
- One comment suggested that we expressed concerns about using self-report data, particularly the finding that music was not considered more important for fostering commitment during the maintenance phase. We would like to clarify that we did not intend to suggest that self-report data was problematic. Instead, we contrasted the quantitative findings with the qualitative insights from the open-ended responses. This has been clarified in the revised text.
- Limitations: In addition to noting the composition of our sample as a limitation, we provided a brief discussion of how the results could have differed with a different composition of the sample, such as including more males, older individuals, or individuals who are less musically inclined. Regarding musical background, we still believe the sample was diverse, as 57% identified as non-musicians and the rest as amateur or semi-professional musicians. Please note that the average of 15 years of musical experience was calculated only for the 61% of the sample who reported playing an instrument or singing.
- As recommended, we added the value of combining the quantitative data with qualitative data in the section “the present study”
- Conclusion: We deleted the note on the access-awareness-agency model from the discussion but added a sentence about it to the introduction, discussing how it helps contextualize our research.

This concludes the changes that we made in response to your comments. We remain open to incorporation of any additional feedback you may have.

# Reviewer report: Reviewer response for version 1

**DOI:** 10.21956/openreseurope.20577.r49262

**Published:** 2025-02-06

**Authors:** Andrea McGraw Hunt

**Affiliations:** 1 Rowan University, Glassboro, New Jersey, USA

The authors conducted a thorough investigation grounded in available theory and evidence. The mixed-methods approach helped explicate the relationships between quantitative measures of musical reward, self-ratings of the importance of music during different stages of romantic relationships, and subjective personal experiences. The qualitative analysis of the open-ended responses appears to be robust and replicable. The authors also made the source data and materials publicly available, a benefit to future researchers for developing follow-up studies.

 Regarding Figure 4, the code cluster for “1 Memories” is categorized as one of the Action Codes, whereas the other codes in this category describe specific actions such as “sharing” or “listening.”  Does “Memories” relate to “making memories”? To me, it seems memories (and even “making” them) is more of an outcome rather than an action. If there’s another explanation, I am curious about it.

 The limitations named by the authors are important. They summarized the demographic characteristics of the sample on page 5, showing the range of gender, age, nationality, and educational level. The demographic questions did not address other cultural or ethnic identities that may have impacted the responses. To check to see if such questions were asked, I looked in the source data at the code book, and discovered there that gender was reduced to a binary code for correlational analyses; the authors did not describe how they handled these data for the 4 individuals who identified as a gender other than male or female. Thus there should be more transparency and clarity for the reader, especially given the otherwise thorough statistical reporting.  Regarding the limitation of selecting participants in different phases of a romantic relationship, perhaps the authors could also suggest recruiting participants involved in polyamorous relationships who may be experiencing long-term committed partnerships alongside newer relationships, and in different configurations. While this may add additional complexity to data collection, the concurrent experiences of the role of music in these relationships could provide valuable subjective reports on the different roles of music in these relationships.

 Overall, this is a robust manuscript with much to offer regarding understanding ways humans engage in music experiences for social bonding. I believe it will provide a rich foundation for further study.

If applicable, is the statistical analysis and its interpretation appropriate?

I cannot comment. A qualified statistician is required.

Is the study design appropriate and is the work technically sound?

Partly

Is the work clearly and accurately presented and does it engage with the current literature?

Yes

Are the conclusions drawn adequately supported by the results?

Yes

Are sufficient details of methods and analysis provided to allow replication by others?

Partly

Are all the source data and materials underlying the results available?

Yes

Reviewer Expertise:

I am a board-certified music therapist with expertise in a wide range of qualitative and quantitative methodologies, particularly neuroscience and chronic pain. I have supervised graduate research involving survey design.

I confirm that I have read this submission and believe that I have an appropriate level of expertise to confirm that it is of an acceptable scientific standard, however I have significant reservations, as outlined above.

# Response

**Published:** 2025-10-22

**Authors:** Julia Vigl

**Affiliations:** Psychology, Universitat Innsbruck Fakultat fur Psychologie und Sportwissenschaft, Innsbruck, Tyrol, Austria

Dear Dr McGraw Hunt,  Thank you very much for taking the time to review our article, and for your positive feedback. We are glad that you appreciated our mixed-methods approach, qualitative analysis and decision to make the data openly available. We have submitted a revised version of the manuscript, which should be available online soon. Below, we summarise the issues you raised and how we addressed each comment, along with the corresponding changes to the manuscript.

1. Thank you for pointing out that the **theme '1 Memories'** could be categorised under the 'outcome' dimension rather than the 'action' dimension. During the coding process, we discussed this issue and decided to place memories in the action dimension because, compared to other outcomes that are more directly tied to emotional or relationship-related processes, we felt that recalling or creating memories functioned more as an active process that could lead to such outcomes. However, we can see how the current wording may be confusing, and therefore have added an explanation of this decision in the Qualitative Results section (right after mentioning Figure 2) and reworded the theme as '1 Making and recalling memories' in the text, figures (Figure 2, Figure 3, Figure 5), and tables (both in the manuscript and in the extended data). 
2. Regarding your comment on **demographics and non-binary participants**: We agree that including questions about cultural or ethnic identity and about the type of relationship (e.g., polyamorous relationship) could have provided valuable context, and we will bear this in mind for future surveys. As these questions were not included in our survey, we have added a new paragraph to the ‘Limitations and future directions’ section. 
3. For gender, in the original manuscript we created a **binary variable for correlational analyses**, coding “female” versus “non-female” (including male, non-binary, and other participants). We recognise that this could be misleading as it might suggest a male–female comparison. In the revised manuscript, we have excluded the four non-binary/other participants from the gender correlations only, while retaining them in all other analyses. The R code in the extended data has been updated accordingly, and the correlation results remain largely unchanged. This approach is now reported in the Data Analysis (quantitative analysis) section.
4. Please note that Table 1 (now labelled Table 2 in the revised manuscript) has been updated in response to comments from the second Reviewer. **Rather than presenting a full correlation matrix, it now only shows the correlations relevant to answering RQ2**, with Benjamini-Hochberg corrections applied for multiple comparisons.

Thank you again for your help in improving this manuscript.
